# Supplementary material for: Influence of the SARS-COV2 pandemic on access to healthcare services among people living with HIV and its subsequent effects on antiretroviral therapy uptake in Malawi
Source: PLOS Glob Public Health. 2024 Sep 5;4(9):e0003665. doi: 10.1371/journal.pgph.0003665 (PMC11376569; doi:10.1371/journal.pgph.0003665)
Supplement: S1 Text — (DOCX) [file pgph.0003665.s001.docx]

**S1_ PLHIV COVID 19 SYNDROMIC SURVEILLANCE QUESTIONNAIRE**

ID 🗆🗆🗆🗆🗆 Date: 🗆🗆/🗆🗆🗆/🗆🗆

Day Month Year

**SCREENING FOR INTERVIEW**

**SOCIO DEMOGRAPHIC HISTORY**

|  |  |
| --- | --- |
| 1. What is your age?...................................................... | 🗆🗆 |
| 1. What is the participant’ sex?....................... | 🗆Male 🗆Female |
| 1. If female what is the pregnancy/breastfeeding woman status of the participant | 🗆Pregnant  🗆Not Pregnant  🗆Breastfeeding  🗆N/A |
| 1. In which district are you currently located? 2. In which traditional authority in Malawi are you currently located |  |
| 1. Which Health facility do you normally get your treatment?....................... | _______________ |

**HISTORY OF COVID 19 SYMPTOMS**

| 1. In the past two weeks, have you been sick or just not feeling well 🗆Yes 🗆No 2. Have you had any of the following new signs and symptoms in the past two weeks? | |
| --- | --- |
| 8a: Fever or feeling feverish?......................................................... | 🗆Yes 🗆No |
| 8b: Persistent Cough?........................................................................... | 🗆Yes 🗆No |
| 8c: Headache?........................................................................... | 🗆Yes 🗆No |
| 8d: Shortness of breath/difficulty breathing? ………..……………………. | 🗆Yes 🗆No |
| 8e: Runny or stuffy nose? ………..……………………. | 🗆Yes 🗆No |
| 8f: Sore throat?.............................................................................. | 🗆Yes 🗆No |
| 8g: Diarrhea?………………………………………. ............................................. | 🗆Yes 🗆No |
| 8h: Muscle body aches? …………………………………………………. | 🗆Yes 🗆No |
| 8i: Lethargy/tiredness…………………………………………………………. | 🗆Yes 🗆No |
| 8j: Loss of smell/taste………………………………………………………… | 🗆Yes 🗆No |
| 8k: Chills………………………………………………………… | 🗆Yes 🗆No |

| 8l: Other Respiratory Symptoms ______________________   \| 1. Did any of these symptoms cause you to go to the hospital/health facility? \| 🗆Yes 🗆No 🗆N/A \| \| --- \| --- \| \| 1. Did any of these symptoms cause you to miss work or school? \| 🗆Yes 🗆No 🗆N/A \| \| 1. How many days since the onset of any of these symptoms (**Range 0-14 days)?.........** 2. Have you had a test for corona virus in the past two weeks? \| 🗆🗆  🗆Yes 🗆No \| \| 1. If yes what were the test results \| 🗆Negative 🗆Positive  🗆Unknown 🗆N/A \| |
| --- | --- | --- | --- | --- | --- | --- | --- | --- |
|  |
| **HISTORY OF HOUSEHOLD ILLNESS / DEATH** |

| 13. Is there anyone in your household aged >60? 🗆Yes 🗆No  14. In the past two weeks, has any members of your household had the following events: | |
| --- | --- |
| 14a: fallen ill with influenza like illness: State a few influenza-like symptoms (Cough, Fever, chillis, runny nose etc.) | 🗆Yes 🗆No |
| 14b: been hospitalized | 🗆Yes 🗆No |
| 14c: put into staying at home alone or away from other people in the community and family due to symptoms of corona virus | 🗆Yes 🗆No |
| 14d: put self to be home alone or away from other people in the community and family (quarantine) due to exposure to corona virus | 🗆Yes 🗆No |
| 14e: tested positive for corona virus | 🗆Yes 🗆No |
| 15. Since January 2020 has any member of your household die from any cause?  ***If no skip to question 18*** | 🗆Yes 🗆No |

**Please let me know if it is okay to ask about the household member who died. *(if participant says it is okay proceed, if not skip to question 20)***

| 16. When did he/she die? …………………………………………… | (DD/MM/YYYY) |
| --- | --- |
| 17. What was the sex of the deceased?....................... | 🗆Male 🗆Female |
| 18. How old was the deceased …………………………………... | _________________ |
| 19. What kind of symptoms in the two weeks prior to his/her death did he have? (select multiple) | |
| 19a: Fever or feeling feverish (chills)?........................................................ | 🗆Yes 🗆No |
| 19b: Sweating?........................................................................................ | 🗆Yes 🗆No |
| 19c: Shortness of breath/difficulty breathing? ………..…………………. | 🗆Yes 🗆No |
| 19d: Cough?............................................................................................... | 🗆Yes 🗆No |
| 19e. None of the above | 🗆Yes 🗆No |
| 20a. Did the deceased test for Corona Virus? | 🗆Yes 🗆No |
| 20b. If yes in (20a), what was the testing result? | 1. 🗆Pos 🗆Neg   🗆Unknown 🗆 N/A |

**MOVEMENT**

| 21. In the past two weeks, have you travelled outside your village/TA? …….…… | | 🗆Yes 🗆No | |
| --- | --- | --- | --- |
| 21a. If yes, on 21c, did you travel outside Malawi?  ***If no in 22 skip to question 24*** | | 🗆Yes 🗆No | |
| 22. Reasons for leaving your home in the past week [select all that apply].? | | | |
| 22a: To go to work or business/college............................................................................. | | 🗆Yes 🗆No | |
| 22b: To get medication.......................................................................... | | 🗆Yes 🗆No | |
| 22c: To get food........................................................................................ | | 🗆Yes 🗆No | |
| 22d: To visit friends and family................................................................... | | 🗆Yes 🗆No | |
| 22e: To provide care for someone.......................................................... | | 🗆Yes 🗆No | |
| 22f: To attend church .......................................................... | | 🗆Yes 🗆No | |
| 22g: To attend a wedding/funeral .......................................................... | | 🗆Yes 🗆No | |
| 22h To attend a political rally/political meeting | | 🗆Yes 🗆No | |
|  | |  | |
| 23i: Other specify  **ACCESS TO HEALTH SERVICES** | | | |
| 24. Has the coronavirus pandemic led to any problems with accessing medical care for anyone within the household?  **If yes** what were the related problems? | 🗆Yes 🗆No | |  |
| 24a. Because it was closed | 🗆Yes 🗆No🗆 N/A | |  |
| 24b. Because I didn’t have transportation | 🗆Yes 🗆No🗆 N/A | |  |
| 24c. Because transportation was not available due to travel restriction | 🗆Yes 🗆No🗆 N/A | |  |
| 24d. Because I couldn’t afford to go to the health facility | 🗆Yes 🗆No🗆 N/A | |  |
| 24e. Afraid travel to the facility as it was against government regulation | 🗆Yes 🗆No🗆 N/A | |  |
| 24f Afraid of getting COVID 19 at the health facility | 🗆Yes 🗆No🗆 N/A | |  |
| 24g. Unable to obtain medications due to stock out | 🗆Yes 🗆No🗆 N/A | |  |
| 24h. Unable to obtain medications due to stock out | 🗆Yes 🗆No🗆 N/A | |  |
| 24i. Other |  | |  |

| 25. In the past week, have you missed any doses of ARVs because you were unable to obtain a medication refill?………  ***If no in 25 above skip to question 29*** | 🗆Yes 🗆No |
| --- | --- |
| 25. **If yes:** what prevented you from collecting your refill? | |
| 25a. Lack of transportation ……………………………… | 🗆Yes 🗆No🗆 N/A |
| 25b. Government Travel restrictions due to corona virus… | 🗆Yes 🗆No🗆 N/A |
| 25c Afraid it is against Government social distancing rules………………. |  |
| 25d. Nearby facilities closed………………………………… | 🗆Yes 🗆No🗆 N/A |
| 25e.Health care workers strike/go slow………………………………… | 🗆Yes 🗆No🗆 N/A |
| 25f.Was busy……………………………………………………… | 🗆Yes 🗆No🗆 N/A |
| 25g.Not feeling well…………………………………………………………… | 🗆Yes 🗆No🗆 N/A |
| 25h.I don`t feel safe going to the clinic because of corona virus …… | 🗆Yes 🗆No🗆 N/A |
| 25i.The nurse/clinician/facility cancelled the appointment……………… | 🗆Yes 🗆No🗆 N/A |
| 25j.Was told ARV/supplies are not available………………………… | 🗆Yes 🗆No🗆 N/A |
| 25k.Long lines/queues……………………………………… | 🗆Yes 🗆No🗆 N/A |
| 20l Other |  |
|  |  |
| 1. When was the last time you collected your ARVs?   (in the estimated number of months) | 🗆🗆🗆 |
| 1. Do you still have your ARVs remaining for you to take? 🗆Yes🗆No 2. In the past month, have you wanted to visit a health facility for HIV care but were unable to do so?… 🗆Yes 🗆No   **Perceived future access to ART**   1. When is your next appointment to visit the health facility for ART refill or clinical visit? _____________________DD/MM/YYY 🗆 Don’t know 2. Do you intend going to the health facility for your refill or next clinical visit?🗆Yes🗆No   **31. If no in 30 above, why not?**   \| 31a. Lack of transportation………………………… \| 🗆Yes 🗆No \| \| \| --- \| --- \| --- \| \| 31b. Government Travel restrictions due to COVID 19…… \| 🗆Yes 🗆No \| \| \| 31c Afraid as it is against Government social distancing rules \| 🗆Yes 🗆No \| \| \| 31d. Nearby facilities are closed………………… \| 🗆Yes 🗆No \| \| \| 31e.Health care workers strike/go slow……………… \| 🗆Yes 🗆No \| \| \| 31f.Will be busy………………………………… \| 🗆Yes 🗆No \| \| \| 31j.Not feeling well…………………………………………… \| 🗆Yes 🗆No \| \| \| 31g.h don`t feel safe going to the clinic because of corona virus \| 🗆Yes 🗆No \| \| \| 31i.The nurse/clinician cancelled the appointment……… \| 🗆Yes 🗆No \| \| \| 31j.Was told ARV/supplies are not available……………… \| 🗆Yes 🗆No \| \| \| 31k.Long lines/queues……………………………………… \| 🗆Yes 🗆No \| \| \| 31l. Other \|  \| \| \| 32. Do you own a face mask for use in public settings? \| \| 🗆Yes 🗆No \| \|  \| **33. If yes in 32 above, where did you get it?** \|  \| \| --- \| --- \| \| 33a Made it myself…………………. \| 🗆Yes 🗆No \| \| 33b. Was given to me at my last visit to the ART clinic \| 🗆Yes 🗆No \| \| 33c. Was given to me \| 🗆Yes 🗆No \| \| 33d. Bought for myself \| 🗆Yes 🗆No \|  \| 33d. Other, \|  \| \| --- \| --- \| |  |

**Thank you so much for responding to this interview. Do you have any questions for me before I close the interview?**
