# Supplementary material for: Influence of the SARS-COV2 pandemic on access to healthcare services among people living with HIV and its subsequent effects on antiretroviral therapy uptake in Malawi
Source: PLOS Glob Public Health. 2024 Sep 5;4(9):e0003665. doi: 10.1371/journal.pgph.0003665 (PMC11376569; doi:10.1371/journal.pgph.0003665)
Supplement: Attachment — Submitted filename: Point by Point Response to the Editors Comments.pdf [file pgph.0003665.s002.pdf]

Point by Point Response to the Editors Comments

. In the ethics statement in the Methods, you have specified that verbal consent was obtained. Please provide additional details regarding how this consent was documented and witnessed, and state whether this was approved by the IRB.

Response: We have provided this detailed and confimed that the verbal consenting process approved by all the IRBs. Please see this statement the edits under the **Ethics Consideration** heading of the manuscript that states the following "***Ethical Considerations***

The study was approved by the Malawi National Health Sciences Research Committee and Advarra Institutional Review Board (IRB) (Protocol #20/06/2537). The Centers for Disease Control and Prevention reviewed and approved this project and determined that it would comply with all requirements of surveillance research. Verbal informed consent approved by all the above IRBs, was obtained from all study participants before conducting interviews to ensure that ethical standards were met throughout the study. As part of verification of the participant's name, as recorded in the electronic medical records system, the initiation of the consenting process ensured that the call respondent were the ones registered as HIV positive client enrolled on ART in a specified EGPAF supported facility. Furthermore, the client was asked if he/she is comfortable speaking about his/her health issues at the time of the call."

2. We note your current Data Availability statement is:

"The data supporting the findings of this study are available upon request from the corresponding author, Thulani Maphosa (email: tmaphosa@pedaids.org). Due to privacy and confidentiality considerations, access to the raw data is restricted, as it contains potentially identifying participant information. However, aggregated data and summary statistics can be made available to interested researchers upon request, subject to appropriate ethical and legal approvals.

In accordance with PLOS Data Policy, we affirm that all relevant data are provided within the manuscript and its Supporting Information files. Any additional inquiries regarding data availability can be directed to the corresponding author."

Do you confirm all relevant data necessary for replicating the study's results is publicly available in the manuscript and Supporting Information files per PLOS's data policy? This does not mean all of the raw data, just the "minimal data set" which consists of the data used to reach the conclusions drawn in the manuscript with related metadata and methods, and any additional data required to replicate the reported study findings in their entirety. For more information, please see https://journals.plos.org/plosone/s/data-availability.

Additionally, could you please provide a non-author point of contact who can field data inquiries from fellow researchers. PLOS does not allow authors to be the sole contact for data inquiries.

We update your Data Availability statement to reflect the information you provide.

Response: In manuscript we have added the data access statement that states that: "**Data *Availability Statement***

All data underlying the findings reported in the manuscript has already been provided as part of the submitted article."

This statement has also been added to the data submission tab.
